# The Relation Between Imposter Phenomenon and Self-Critical, Narcissistic, and Rigid Perfectionism: An Observational Study from a Women’s Medical College in Saudi Arabia

**DOI:** 10.3390/healthcare13243311

**Published:** 2025-12-17

**Authors:** Nasser M. AbuDujain, Rauof A. Almebki, Rakan M. Alghonaim, Mohammed A. Aldkhyyal, Norah A. Alshehri, Saud Alomar, Ahmed S. Almujil, Joud S. Almutairi, Msaad A. Altulihee, Turky H. Almigbal

**Affiliations:** 1 Department of Family and Community Medicine, College of Medicine, King Saud University, Riyadh 11495, Saudi Arabia; rakanalghonaim@gmail.com (R.M.A.); drnora@ksu.edu.sa (N.A.A.); dr.jouds@hotmail.com (J.S.A.); maltulihee1@ksu.edu.sa (M.A.A.); 2 College of Medicine, Princess Noura bint Abdulrahman University, Riyadh 11564, Saudi Arabia; famobki@gmail.com; 3 College of Medicine, King Saud University, Riyadh 11451, Saudi Arabia; mohammedaldkhyyal@gmail.com; 4 Pediatric Department, King Abdullah bin Abdulaziz University Hospital, Riyadh 11671, Saudi Arabia; drpeds2010@gmail.com; 5 Family Medicine & Community Health Department, King Abdullah bin Abdulaziz University Hospital, Riyadh 11671, Saudi Arabia; dr.aalmujil@yahoo.com

**Keywords:** imposterism, imposter phenomenon, perfectionism, self-efficacy, medical students

## Abstract

**Background/aim:** Medical students often face intense pressure to excel, which can lead to imposterism, characterized by persistent self-doubt and fear of being exposed as inadequate, alongside high levels of perfectionism. This study aims to assess the prevalence of imposterism and perfectionism among medical students in Saudi Arabia and explore their potential implications for student well-being. **Methods:** A cross-sectional analytical study was conducted between October and December 2024 among female medical students in Saudi Arabia. Data were collected via an online survey, which included demographic questions, the validated Arabic versions of the Clance Impostor Phenomenon Scale (Ar-CIPS), the short form of the Big Three Perfectionism Scale (BTPS-SF), and the General Self-Efficacy Scale (GSES). **Results:** A total of 265 medical students (mean age 20.96 ± 1.44 years) participated. Most reported a monthly income of less than 2000 SR, and the majority had a GPA above 4.5. A 74.3% expressed moderate impostor experiences. The BTPS-SF revealed the highest scores in self-critical perfectionism, followed by narcissistic and rigid perfectionism. Higher impostor scores were significantly associated with greater perfectionism and lower self-efficacy (*p* < 0.05). In multivariable analysis, BTPS-SF scores positively predicted CIPS scores (β = 0.52, *p* < 0.001), whereas GSE scores predicted lower CIPS scores (β = −0.47, *p* = 0.001). **Conclusions:** This study revealed a high prevalence of impostor phenomenon and perfectionism among female medical students, with self-critical perfectionism being the most prominent. Imposterism was significantly associated with higher perfectionism and lower self-efficacy.

## 1. Introduction

When it comes to medical students, the daunting feeling and pressure to be a high achiever with high competencies leads to self-doubt, low self-esteem and something called imposterism [1]. This phenomenon, often called imposter syndrome, describes individuals who persistently doubt their accomplishments and have feelings of self-doubt in fear of being perceived as fraudulent [2]. Individuals with this phenomenon often struggle to accurately associate their performance with their full potential and competency [3]. A study conducted among medical students in the United States showed a significant correlation between IS and burnout indicators like exhaustion and depersonalization [4]. A recently published article in 2024 by Wrench et al. identified imposterism in 40% of first-year medical students, with 84% being females [5]. Another study conducted among matriculating first-year medical students found that 87% of students reported high or very high levels of imposterism; this percentage increased by the end of the year [6].

Perfectionism has been a topic of widespread interest; it has been widely studied and discussed throughout the 20th century; literature has established adaptability to this behavior [7]. Psychologists define perfectionism as “a personality trait characterized by a person’s striving for flawlessness and setting exceedingly high-performance standards, accompanied by critical self-evaluations and concerns regarding others’ evaluations” [8]. In 1991, Hewitt and Flett identified three dimensions of perfectionism: self-oriented, other-oriented, and socially prescribed [9]. Perfectionists engage in behaviors to please themselves and others or avoid being socially ridiculed [7]. A recent cross-sectional study conducted among 489 undergraduate medical students in Saudi Arabia explored the prevalence of perfectionism and its relationship to academic achievement; findings suggest that perfectionism is prevalent among undergraduate medical students and that self-oriented perfectionism is the most common subtype [8]. A nuanced conceptualization of perfectionism has created a split in the literature. While in the past, researchers spent time studying the maladaptive aspect of perfectionism and its negative consequences on a person’s well-being, more recent studies found that it can positively influence mental health [10]. In 2020, a literature review provided valuable insight into the prevalence and impact of perfectionism among medical students. In comparison to other student groups, medical students exhibited lower maladaptive perfectionism scores, though their adaptive perfectionism scores were not significantly different [11].

Despite several studies exploring the prevalence of impostor phenomenon and perfectionism, these constructs remain understudied among medical students in Saudi Arabia. Understanding their prevalence and interrelationships is essential, as they may influence students’ psychological functioning and overall well-being, even though the present study does not directly measure mental health outcomes. Therefore, this study aimed to assess the prevalence of the impostor phenomenon and perfectionism among female medical students and to examine how these constructs relate to self-efficacy within the context of a large governmental women’s medical institution. Based on prior literature, we hypothesized that (1) higher levels of perfectionism—particularly self-critical perfectionism—would be associated with higher impostor phenomenon scores, and (2) higher levels of general self-efficacy would be associated with lower impostor phenomenon scores. By addressing these relationships, this study contributes important evidence to an area that remains insufficiently explored in the region.

## 2. Methodology

### 2.1. Study Design, Participants, and Setting

A quantitative analytical cross-sectional study was carried out targeting female students of the College of Medicine at Princess Nourah Bint Abdulrahman University (PNU), Riyadh, Saudi Arabia. The survey was conducted between October and December 2024. The sample size was determined based on the total population of 495 medical students from the 1st to 5th year at PNU. Using a 95% confidence level and a 5% margin of error, the initial sample size was calculated using the standard formula for proportions. A finite population correction was then applied, resulting in a required minimum sample size of 217.

### 2.2. Questionnaire

The survey created for this study consisted of a demographic and academic information section, the Clance IP scale, the Big Three Perfectionism Scale—short form and the General self-efficacy scale, with the latter three aiming to survey how students perceived impostor phenomenon and perfectionism and self-efficacy in themselves.

#### 2.2.1. Personal and Academic Information

This section inquired about demographic information, including age, monthly personal income, and number of family members. Following that, academic information asked about the current academic year and students’ grade point average (GPA) out of 5.

#### 2.2.2. Clance Imposter Phenomenon Scale (CIPS)

The Clance Impostor Phenomenon Scale (CIPS) was originally developed by Pauline R. Clance in 1985 to assess the core dimensions of the impostor phenomenon: (a) fear of evaluation, (b) fear of being unable to sustain success, and (c) fear of being less competent than others. The scale comprises 20 self-reported items rated on a 5-point Likert scale ranging from 1 “Not at all true” to 5 “Very true”. Total scores range from 20 to 100, with interpretive thresholds as follows: ≤40 indicates few impostor characteristics, 41–60 suggests moderate impostor experiences, 61–80 reflects frequent impostor feelings, and scores above 80 denote severe impostor experiences. Higher scores correspond to greater interference of impostor feelings in an individual’s life [12]. In this study, we utilized the validated Arabic version of the CIPS (Ar-CIPS), developed by AbuDujain et al. in 2025 [13]. The Ar-CIPS demonstrated strong psychometric properties, with a Cronbach’s alpha of 0.90 and an intraclass correlation coefficient of 0.71, indicating excellent internal consistency and test–retest reliability. Furthermore, the scale exhibited robust discriminant and structural validity.

#### 2.2.3. Big Three Perfectionism Scale (BTPS)—Short Form

The Big Three Perfectionism Scale (BTPS), developed by Smith et al. in 2016 [14], is a 45-item multidimensional measure designed to assess ten facets of perfectionism, which are grouped into three comprehensive factors: rigid perfectionism, self-critical perfectionism, and narcissistic perfectionism. In 2019, Feher et al. [15] developed a shortened 16-item version of the BTPS by selecting one or two items per facet based on the highest factor loadings and minimal cross-loadings, while retaining the original three-factor structure. Items are rated on a 5-point Likert scale ranging from 1 “Strongly disagree” to 5 “Strongly agree”. In this study, we utilized the validated Arabic version of the BTPS-SF, developed by Fekih-Romdhane et al. in 2023 [16]. The Arabic BTPS-SF demonstrated strong psychometric properties, with Cronbach’s alpha and McDonald’s omega coefficients ranging from 0.83 to 0.86, indicating excellent internal consistency [14,15,16].

#### 2.2.4. General Self-Efficacy Scale (GSES)

The General Self-Efficacy Scale (GSES), developed by Schwarzer and Jerusalem in 1995, assesses an individual’s belief in their ability to cope with challenging situations and stressful life events. The scale comprises 10 items, each rated on a 4-point Likert scale ranging from 1 “Not at all true” to 4 “Exactly true”, with total scores ranging from 10 to 40. Higher scores indicate greater perceived self-efficacy. In our study, the Arabic version of the GSES was employed, it was validated back in 2017, demonstrating excellent internal consistency with a Cronbach’s alpha of 0.95, which confirms its reliability and cultural relevance among Arabic-speaking populations [17,18].

### 2.3. Procedure, Ethical Consideration and Data Collection

The ethical review board in the King Saud University has approved this study (approval code: E-24-8787, Ref. No. 24/1310/IRB, approval date: 7 May 2024), this study was performed using an online survey via the SurveyMonkey website, and the survey was distributed using a link sent to participants, the nature and purpose of the study, the primary investigator’s contact information, and an explanation of the confidentiality and data anonymity policy were provided. Consent to participate was given by clicking on the informed consent link. After reading the informed consent statement, the participants clicked “Next” to access this study’s survey, which took approximately 5–7 min to complete.

### 2.4. Statistical Data Analysis

Statistical analysis was performed by SPSS version 28 (IBM Co., Armonk, NY, USA). Numerical data were presented as the mean and standard deviation (SD) and analyzed using One-way ANOVA. Categorical data were presented as frequency and percentage. Pearson’s correlation coefficient was calculated to estimate the degree of correlation between two quantitative variables. Linear regression analysis was performed to assess factors associated with the CIPS score. A two-tailed *p*-value < 0.05 was considered statistically significant.

## 3. Results

This study was conducted on 265 medical students (All females) with a mean age of 20.96 ± 1.44 years. More than two-thirds of participants (74.3%) reported having less than 2000 SR as their monthly income. As regards GPA, it was between 4.5 and 4.74 for 27.5% of students and between 4.75 and 5 for 47.5%. Moreover, 20.4%, 20%, 19.2%, 19.2% and 21.1% of students were in their 1st, 2nd, 3rd, 4th and 5th year, respectively (Table 1).

Regarding CIPS, participants gave the highest scores for the statements “I feel bad and discouraged if I’m not “the best” or at least “very special” in situations that involve achievement”, “I often compare my ability to those around me and think they may be more intelligent than I am”, “I’m disappointed at times in my present accomplishments and think I should have accomplished much more” and “I have often succeeded on a test or task even though I was afraid that I would not do well before I undertook the task”, respectively, with a mean score of 3.58 ± 0.95, 3.44 ± 0.98, 3.27 ± 1.03 and 3.26 ± 0.85. Overall, the mean total score was 55.18 ± 10.42, with 74.3% of participants exhibiting moderate IP experiences.

As for BTPS-SF, the highest score was for self-critical perfectionism (with a mean of 16.8 ± 4.12), followed by narcissistic perfectionism (14.85 ± 3), then rigid perfectionism (10.91 ± 2.99), with an overall score of 42.55 ± 7.83. GSE scale showing that the statement “I can solve most problems if I invest the necessary effort” gained the highest score, with a mean of 3.29 ± 0.56, followed by the statements “I can always manage to solve difficult problems if I try hard enough” and “If I am in trouble, I can usually think of a solution”, with a mean of 3.21 ± 0.63 and 3.2 ± 0.62, respectively. The mean total GSE score was 27.83 ± 4.27. Pearson’s correlation analysis revealed a significant positive correlation between CIPS and BTPS-SF scores (r = 0.467, *p* < 0.001), as well as significant negative correlations between GSE and CIPS (r = −0.270, *p* < 0.001) and BTPS-SF (r = −0.167, *p* = 0.007).

There was a statistically significant relation between the frequency of IP experiences and BTPS-SF scores (*p* < 0.05); participants with intense IP characteristics manifested significantly higher rigid perfectionism scores than those with few, moderate and frequent IP experiences. The self-critical perfectionism score was significantly higher among subjects with intense IP than among those with frequent IP, and was significantly higher in both groups than among those with few and moderate IP experiences. As for the narcissistic perfectionism score, it was significantly higher in the case of moderate than in the case of a few IP experiences. The total BTPS-SF score was significantly higher among participants with intense IP than those with moderate and frequent IP, and was significantly higher in subjects with frequent and intense IP experiences than those with few experiences. Additionally, a significant relationship was observed between IP and GSE (*p* < 0.001), with significantly lower GSE scores among participants who experienced frequent and intense IP compared to those with few and moderate IP experiences (Table 2).

In the univariate linear regression analysis, most demographic variables—including age, monthly income, GPA, and academic year—were not significantly associated with total CIPS score. The only significant factors in the univariate analysis were the BTPS-SF and GSE scores. Each one-unit increase in BTPS-SF score was associated with a significantly higher CIPS score (coefficient = 0.62, 95% CI: 0.48 to 0.76, *p* < 0.001). Conversely, each one-unit increase in GSE score was significantly associated with a lower CIPS score (coefficient = −0.66, 95% CI: −0.95 to −0.37, *p* < 0.001). In the multivariable model, after adjusting for age, income, GPA, and academic year, only BTPS-SF and GSE scores remained statistically significant. Higher BTPS-SF scores continued to be associated with higher CIPS scores (coefficient = 0.58, 95% CI: 0.42 to 0.74, *p* < 0.001), whereas higher GSE scores were independently associated with lower CIPS scores (coefficient = −0.45, 95% CI: −0.73 to −0.18, *p* = 0.001). No other variables retained statistical significance in the multivariable model (Table 3).

The selection of covariates for the regression models was guided by theoretical relevance and prior literature on academic and psychological correlates of impostor phenomenon, with particular emphasis on academic performance (GPA), socioeconomic status (monthly income), and training stage (academic year) as potential structural confounders. Conceptually related constructs as perfectionism (BTPS-SF) and general self-efficacy (GSE)—were incorporated. Assumptions of linear regression were evaluated. Multicollinearity was assessed using variance inflation factors (VIF), with all VIF values < 2, indicating no evidence of collinearity among predictors. Normality of residuals and homoscedasticity were examined using residual plots and were deemed acceptable for linear modeling.

## 4. Discussion

This study explores the frequency of IP and perfectionism in 265 female medical students at PNU and examines their links with self-efficacy. The results show a high presence of marked self-critical perfectionism, or moderate to high self-efficacy and an association among these constructs. As a result of these findings, we argue that major psychological pressures in medical education are more pronounced and concentrated in a single sex group, PNU’s medical students, within Saudi Arabia’s cultural context.

The prevalence of moderate IP seen in this study (74.3%) is remarkably high compared to elsewhere. For example, Elnaggar et al. reported that 50.3% of Saudi medical students have frequent moderate internalizing perfectionism, while Alrefi et al. noted that 49.4% of Saudi medical and nursing students attain high or intense IP, both using CIPS [1,19]. Internationally, Kristoffersson et al. indicated 58.4% of Swedish medical students hold IP over themselves, and Naser et al. found Middle Eastern peers also at a rate of 45.2% [20,21]. Rates downward are seen in Pakistan (55.74%), India (48.5% frequent/high intensity) and the most likely Western setting of Wrench et al., where it fell to 40% using YIS [5,22,23]. In scenario, Alrefi et al. and Naser et al. reported that, alongside their female surgical peers, it is of concern for female medical students in Saudi Arabia, a country long known for both intense cultural pressures to excel and its society-wide emphasis on the best results. It is said that besides equally punctilious scores on “when I experience a setback,” high rankings for “cannot tolerate making mistakes” and “I have to be the top” seem to put internalized unrealistic standards. This is consistent with earlier findings by Jurkowski et al., that IP involves continual self-doubt which is eventually invested in academic performance [24]. Each case designed an alliance with systemic psychotherapy. Importantly, classification within the “moderate” impostor phenomenon range reflects more than a numerical threshold. A mean CIPS score of approximately 55 corresponds to recurrent self-doubt, heightened sensitivity to evaluation, and difficulty internalizing success despite objective achievement. In the context of medical education, such experiences may manifest as over-preparation, reluctance to engage confidently in academic or clinical discussions, persistent fear of underperformance, and challenges in day-to-day academic decision-making. Thus, although labeled as “moderate,” these impostor experiences may represent a chronic cognitive-emotional burden that subtly interferes with learning, confidence development, and professional identity formation, particularly in high-stakes training environments.

The average BTPS-SF score in our sample (42.55 ± 7.83), driven primarily by the self-critical subscale (16.8 ± 4.12), reflects a substantial level of maladaptive perfectionism. This pattern is consistent with evidence demonstrating that self-critical and concern-oriented dimensions of perfectionism are closely associated with elevated impostor feelings in academically demanding contexts [25]. Perfectionistic tendencies—particularly unrealistically high personal standards and heightened sensitivity to mistakes—have also been shown to intensify the self-doubt and competence-related concerns characteristic of the impostor phenomenon and may contribute to increased psychological distress when combined with the competence doubts central to imposterism [26,27]. In the present study, the significant positive correlation between CIPS and BTPS-SF scores supports the view that perfectionism amplifies impostor-related cognitions by promoting internal expectations that are difficult to attain [25]. Participants reporting stronger impostor feelings also demonstrated higher levels of self-critical perfectionism, suggesting that maladaptive perfectionistic patterns may heighten perceptions of inadequacy and inauthenticity within rigorous medical training environments [27]. From an applied educational perspective, the observed relationships between perfectionism, impostor phenomenon, and self-efficacy offer actionable implications for curriculum design. The inverse association between self-efficacy and imposterism suggests that interventions aimed at strengthening perceived competence such as structured formative feedback, mentorship programs, mastery-oriented assessment strategies, and reflective learning opportunities may help mitigate impostor experiences. Simultaneously, addressing maladaptive self-critical perfectionism through cognitive-behavioral or skills-based workshops may reduce the internal pressures that sustain impostor feelings. These findings indicate that fostering self-efficacy and moderating perfectionistic tendencies concurrently, rather than sequentially, may be particularly beneficial within medical training programs.

The mean GSES score in our sample (27.83 ± 4.27) suggests moderate to high self-efficacy, indicating that students generally perceive themselves as capable of managing academic challenges. The negative correlations observed between GSES, CIPS, and BTPS-SF support the view that higher self-efficacy serves as a protective factor against both impostor feelings and maladaptive perfectionism [25]. This relationship is consistent with prior evidence showing that lower self-efficacy contributes to stronger impostor tendencies and exacerbates self-doubt [25,28]. Further reinforcing this pattern, previous research has demonstrated a significant negative correlation between CIPS and GSES, with increases in self-efficacy predicting meaningful reductions in impostor scores—for example, a one-point increase in GSES was associated with a 0.47-point decrease in CIPS [20]. Collectively, these findings underscore the central role of self-efficacy in mitigating impostor-related cognitions and help explain why students with stronger perceived capability exhibit lower levels of imposterism in demanding academic programs such as medicine [20]. As observed in other fields examining interrelated but distinct constructs, correlated variables do not necessarily reflect a single underlying dimension. For example, Cesare et al. reported that although medical complexity and nursing complexity of care were associated, the frequency of nursing actions did not directly correspond to the number of chronic conditions, illustrating that related indicators can represent separate facets of a phenomenon [29]. In the present study, although perfectionism, impostor phenomenon, and self-efficacy were significantly correlated, they reflect conceptually distinct psychological processes rather than interchangeable or causally ordered elements. Therefore, the observed associations should be interpreted as coexisting dimensions of individual differences rather than evidence that one construct subsumes or explains the others in a causal hierarchy.

In our study, all participants were female, a feature that aligns with broader evidence indicating that women consistently report higher levels of impostor feelings than men [20,23,25]. This pattern has been documented across multiple cultural and educational contexts, including Middle Eastern settings, where women often face heightened academic expectations and internalized pressure related to professional advancement [19]. The Arabic versions of the psychometric instruments used in this study (Ar-CIPS, BTPS-SF, GSES) have undergone rigorous validation, ensuring their technical adequacy and cultural relevance for assessing IP-related constructs in Arab populations [23]. Research has further demonstrated that academic environments characterized by high performance demands can exacerbate impostor tendencies and perfectionistic self-evaluation [22], structured programs that target cognitive-behavioral skills, peer support, and resilience-building have been identified as promising strategies for mitigating impostor feelings in university settings [5,27]. Healthcare systems and roles are rapidly growing in complexity, requiring clinicians not only to demonstrate traditional clinical competencies but also to adapt to multifaceted leadership, innovation, and collaborative demands in unpredictable environments. Recent scoping work by Spanos et al. underscores how future healthcare leaders must cultivate adaptability, innovation, and self-awareness in response to evolving systemic pressures, suggesting that the psychological load on trainees and early professionals may be compounded by these expanding role expectations [30]. This risk may be further amplified by the increasingly complex nature of contemporary healthcare systems, where the clinician–patient interaction itself has been described as a complex adaptive system. A’aqoulah et al. emphasized that complexity in primary healthcare extends to real-time data analytics and medication prescribing, and argued that future health systems require adaptive leadership, transdisciplinary redesign, robust monitoring and evaluation, and greater patient-safety and information-transparency practices supported by effective health information technology [31]. Within a single-sex medical college, specific contextual factors may further intensify these psychological patterns. Closed academic environments can amplify peer comparison and internal benchmarking, particularly among high-achieving cohorts, reinforcing fears of inadequacy and self-directed perfectionism. Additionally, cultural and gendered expectations surrounding excellence and responsibility may heighten internal pressure to perform flawlessly while minimizing visible struggle. These dynamics may help explain why self-critical perfectionism consistently emerged as the most elevated perfectionism subtype in this cohort, suggesting that impostor experiences are shaped not only by individual traits but also by the educational and sociocultural context in which training occurs.

This study’s strengths lie in three main aspects: an adequately large sample size (*n* = 265), the exclusive employment of validated Arabic scales, and a full analysis of IP, perfectionism and self-efficacy. However, the cross-sectional nature of this research design and its reliance on self-reported data may engender recall bias to limit causal inferences. With the female-only sample, however, it is not clear that we can generalize to a mixed gender setting, though these data do offer some insight into one particular group of people.

## 5. Conclusions

A large proportion of medical students at PNU experience moderate levels of impostor phenomenon, with self-critical perfectionism emerging as the most prominent trait associated with these feelings. Additionally, higher self-efficacy was associated with lower levels of imposterism, suggesting that it may serve as a protective factor.

## 6. Future Directions

We recommend that future researchers further explore how these patterns develop and change over time through longitudinal studies, assess the effectiveness of targeted interventions aimed at improving self-efficacy and reducing perfectionism, and include more diverse samples across universities, majors, and genders to enhance generalizability and better understand the influence of different cultures and backgrounds from people across all walks of life.

## Figures and Tables

**Table 1:** Socio-demographic characteristics of study participants (*n* = 265).

Item	*n*	%
Age (years)		
Mean ± SD	20.96 ± 1.44	
Monthly income (SR)		
<2000	197	74.3
2000–<5000	56	21.1
5000–<10,000	6	2.3
≥10,000	6	2.3
GPA		
3.5–3.99	8	3.0
4–4.49	58	21.9
4.5–4.74	73	27.5
4.75–5	126	47.5
Academic year		
First year	54	20.4
Second year	53	20.0
Third year	51	19.2
Fourth year	51	19.2
Fifth year	56	21.1

**Table 2:** Relation between frequency of IP experiences and BTPS-SF and GSE scores.

Item	CIPS				*p*-Value
	Few IP Experiences	Moderate IP Experiences	Frequent IP Experiences	Intense IP Experiences	
BTPS-SF					
Rigid perfectionism	10.23 ± 2.68 ^a^	10.58 ± 2.34 ^a^	10.96 ± 3.73 ^a^	17.9 ± 2.96 ^b^	<0.001
Self-critical perfectionism	14.85 ± 3.48 ^a^	15.95 ± 3.18 ^a^	18.87 ± 4.6 ^b^	26.7 ± 2.5 ^c^	<0.001
Narcissistic perfectionism	12.62 ± 2.69 ^a^	15.02 ± 2.58 ^b^	14.56 ± 3.47 ^ab^	15.7 ± 6.46 ^ab^	0.028
Total score	37.69 ± 5.75 ^a^	41.55 ± 6.2 ^ab^	44.38 ± 9 ^b^	60.3 ± 9.83 ^c^	<0.001
GSE score	30.85 ± 4.3 ^a^	28.17 ± 3.5 ^a^	26.31 ± 5.6 ^b^	24 ± 6.75 ^b^	<0.001

Numerical data are presented as mean ± SD, Statistical significance at *p*-value < 0.05, Different lower-case letters indicate significant difference in pairwise comparison (a mean differs significantly from groups without a; b mean differs significantly from groups without b; c mean differs significantly from groups without c).

**Table 3:** Linear regression analysis for factors associated with total CIPS score.

Item	Univariate Analysis			Multivariable Analysis		
	Coefficient	95% CI	*p*-Value	Coefficient	95% CI	*p*-Value
Age (years)	−0.05	−0.93 to 0.83	0.911	−0.22	−1.93 to 1.5	0.801
Monthly income (SR)						
<2000	Ref			Ref		
2000–<5000	−1.14	−4.24 to 1.96	0.471	−0.51	−3.39 to 2.36	0.726
5000–<10,000	−1.63	−10.13 to 6.86	0.705	−2.16	−9.77 to 5.45	0.577
≥10,000	7.03	−1.46 to 15.53	0.104	4.41	−3.4 to 12.22	0.267
GPA						
3.5–3.99	Ref			Ref		
4–4.49	−5.67	−13.37 to 2.03	0.148	−3.85	−10.8 to 3.11	0.277
4.5–4.74	−8.06	−15.67 to −0.46	0.038	−6.7	−13.8 to 0.4	0.064
4.75–5	−6.01	−13.45 to 1.44	0.113	−5.54	−13.04 to 1.96	0.147
Academic year						
First year	Ref			Ref		
Second year	−1.55	−5.53 to 2.43	0.443	0.6	−7.59 to 8.78	0.886
Third year	−2	−6.02 to 2.02	0.329	1.2	−5.59 to 7.99	0.727
Fourth year	−2.74	−6.76 to 1.28	0.181	1.42	−4.1 to 6.94	0.613
Fifth year	−1.51	−5.44 to 2.42	0.45	0.68	−3.5 to 4.86	0.75
BTPS-SF score	0.62	0.48 to 0.76	<0.001	0.58	0.42 to 0.74	<0.001
GSE score	−0.66	−0.95 to −0.37	<0.001	−0.45	−0.73 to −0.18	0.001

CI: Confidence interval, Statistical significance at *p*-value < 0.05. Ref means Reference category.

## Data Availability

Data used in this study are available upon reasonable request from the corresponding author due to privacy and ethical restrictions.
